# Melanocytic nevi and melanomas of the oral mucosa: detailed description of a case series^⋆^

**DOI:** 10.1016/j.abd.2024.03.005

**Published:** 2024-08-07

**Authors:** Izadora Fernanda Veiga de Jesus Costa, Deyla Duarte Carneiro Vilela, Bruno Cunha Pires, Jener Gonçalves de Farias, Valéria Souza Freitas, Jean Nunes dos Santos

**Affiliations:** a Dentistry and Health Postgraduate Program, Faculty of Dentistry, Universidade Federal da Bahia, Salvador, BA, Brazil; b Centro de Anatomia Patológica Pires, Feira de Santana, BA, Brazil; c Department of Health, Universidade Estadual de Feira de Santana, Feira de Santana, BA, Brazil; d Laboratory of Oral and Maxillofacial Pathology, Salvador, BA, Brazil

*Dear Editor,*

Oral melanocytic nevi are asymptomatic lesions that can be pigmented or not.^1^, ^2^ Oral melanomas are extremely rare malignant neoplasms^3^ and can also be pigmented and non-pigmented (amelanotic),^4^ a fact that renders their clinical diagnosis challenging. We describe a case series of melanocytic nevi and melanomas located in the oral cavity. They were diagnosed among 4,030 oral biopsies between 2002 to 2021. The most frequent nevus was blue nevus (46.67%, n = 7), followed by compound nevus (26.67%, n = 4) and intramucosal nevus (26.67%, n = 4). Melanomas affected two adult males. The clinical data of the selected cases are shown in Table 1. Table 2 shows the histopathological characteristics of the selected cases. The histopathologic features of nevi and melanomas are represented in Figure 1, Figure 2. The melanocytic nevi studied here showed a female predilection (80%) and were commonly found on the palate (60%) due to cases of blue nevus.^1^, ^2^ Only two intramucosal nevi were located on the palate. Oral nevi are diagnosed on average in the third and fourth decade of life.^1^ According to some authors,^1^ blue nevi are identified in older patients, while compound nevi occur in younger patients, as observed in our study. In general, the lesions identified had a dark or black color and the mean size (about 4 mm). It is important to note that two of the lesions identified did not have a dark color as reported by Buchner et al.;^2^ both of them were diagnosed as intramucosal nevus. No case of junctional nevus was identified in the present study. All of the nevi met showed symmetry,^5^, ^6^ an important feature for defining them as benign neoplasms.^5^ Histologically, the epithelial lining of the nevi displayed hyperparakeratosis/hyperkeratosis which were observed in one case of compound nevus and one case of intramucosal nevus, respectively. Papillomatous/verrucous surfaces accompanied by hyperkeratosis and acanthosis are a common feature in skin nevi.^5^ Pseudoepitheliomatous hyperplasia was observed in only two cases. Melanin was found especially in the basal layer of the epithelial lining of the nevi. This is a usual finding since most of the lesions were black, except for two cases located on the hard palate and lower lip, which were covered with non-pigmented mucosa. Dendritic cells were only seen in two nevi, although the presence of dendritic melanocytes is not surprising in the oral mucosa. The cellular pattern of the nevus was variable, with the cells being frequently arranged in nests or theques, probably corresponding to cases of non-blue nevi. However, the cells were not arranged in a single pattern, with the identification of more than one type in the same lesion, a finding that might be attributed to the process of maturation.^6^ The morphology of nevus cells varies depending on their location and this variation has been associated with their stage of maturation.^6^, ^7^ It was often difficult to identify all cell types (Type A, Type B, Type C cells) specifically and clearly in the same case. However, two cases of intramucosal nevus exhibited all of these cellular elements. In 53.33% (n = 8) of the cases, the cells had a spindle shape and were arranged parallel to the surface, typical findings of blue nevus. Neurotization was found in two cases of intramucosal nevus and one case of compound nevus. Pseudo-inclusions and giant cells were identified in intramucosal nevi, as previously described.^7^ Melanophages also indicate maturation and were found in nine of the selected cases. All of the cases were treated by surgical excision, and no sign of recurrence was detected.^2^ However, in three cases the lesions were not removed completely. None of the cases of nevi had any histological indication or suspicion of malignancy as asymmetry. Melanoma is the main differential diagnosis of these lesions, although pigmentation of exogenous and endogenous origin should also be included. We identified only two cases of invasive melanoma in male patients in the fifth and sixth decade of life, demonstrating that this neoplasm is very rare in the mouth, particularly amelanotic melanoma.^8^ Immunohistochemistry for S100 and melan A was important for diagnostic confirmation and differential diagnosis. The absence of melanin pigment makes the diagnosis of amelanotic melanoma difficult. Extraosseous plasmocytoma was the main differential diagnostic in the case #16 (amelanotic melanoma) whereas the pigmention in the case #17 suggested to be a melanoma although neural neoplasms can show pigment and are included in the differential diagnosis as well. It is important to state that concerning case #16, the patient reported being HIV positive. Melanoma is known to have an unfavorable prognosis and there might be an association with the individual’s immune condition. Previous studies have evaluated the development of melanoma in transplant recipients, patients with non-Hodgkin’s lymphoma, and HIV-positive patients.^9^ HIV-infected patients show immunological alterations that may contribute to the development of other malignant lesions, including the increased production of Th2 cytokines.^10^ However, more studies are needed to elucidate the existence of a relationship between immunosuppression and an increased risk of developing melanoma. This study showed that cellular nevi and invasive melanomas of the oral cavity are rare neoplasms, although none of the nevi exhibited any changes that would indicate malignancy. Therefore, both types of lesions must be included in the differential diagnosis of pigmented and non-pigmented lesions of the oral cavity.

**Table 1:** Clinical data of the 17 selected cases.

Case	Sex	Age (years)	Diagnosis	Size (mm)	Clinical hypotheses	Affected site	Appearance	Color	Excision
1	Female	50	Blue nevus	2	Nevus/melanotic macule	Hard palate	‒	Dark	Complete
2	Female	41	Blue nevus	‒	Nevus/melanotic macule	Hard palate	Macule	Black	Complete
3	Female	34	Blue nevus	‒	Amalgam tattoo	Hard palate	‒	Dark	Complete
4	Female	29	Blue nevus	3	Blue nevus/melanoma	Hard palate	Macule	Black	Complete
5	Female	19	Compound nevus	‒	‒	Cheek mucosa	‒	Dark	Complete
6	Female	29	Intramucosal nevus	‒	Traumatic fibroma	Hard palate	Nodule	Non-pigmented	Complete
7	Male	40	Intramucosal nevus	10	‒	Hard palate	Spot	Black	Incomplete
8	Female	43	Intramucosal nevus	2	Melanotic macule	Lower lip mucosa	Spot	Black	Complete
9	Female	31	Blue nevus	2	Nevus/melanotic macule	Hard palate	‒	‒	Complete
10	Female	30	Blue nevus	‒	Melanotic macule	Hard palate	Macule	‒	Incomplete
11	Female	14	Compound nevus	‒	‒	Cheek mucosa	‒	Black	Incomplete
12	Male	74	Blue nevus	6	Melanotic macule/nevus	Hard palate	Macule	Black	Complete
13	Female	‒	Intramucosal nevus	4	Focal fibrous hyperplasia	Lower lip	Nodule	Normal mucosa-like color	‒
14	Male	51	Compound nevus	‒	‒	Gingiva	‒	‒	‒
15	Female	25	Compound nevus	‒	‒	Upper lip	‒	‒	‒
16	Male	54	Melanoma	30	Pyogenic granuloma	Gingiva and alveolar mucosa	Nodule	Reddish/Grayish	Incomplete
17	Male	44	Melanoma	50	Kaposi sarcoma	Palatal mucosa	Nodule	Black	-

**Table 2:** Histopathological characteristics of the selected cases of melanocytic nevi.

Characteristic	Blue nevus	Intramucosal nevus	Compound nevus
**Epithelial lining**			
Hyperkeratosis	0	0	0
Hyperparakeratosis	1	1	0
Acanthosis	6	2	2
Atrophy	0	3	2
Dendritic cell	1	0	1
Papillomatous/verrucous surface	0	0	0
Presence of melanin	1	0	1
Pseudoepitheliomatous hyperplasia	0	2	1
**Mitosis**			
Upper lamina propria	0	1	0
Lower lamina propria	0	0	0
**Cellular pattern**			
Nests	0	3	2
Cords	0	1	0
Sheets	0	2	2
Other	4	1	0
**Cytoplasm**			
Polygonal	3	2	0
Epithelioid	0	3	2
Spindle-shaped	7	0	0
Clear cytoplasm	0	3	1
Pale cytoplasm	0	0	0
Slightly eosinophilic cytoplasm	0	3	2
Cytoplasm with melanin	7	2	2
Pleomorphism	0	1	1
Hyperchromatism	0	1	1
**Nucleus**			
Rounded to oval, with prominent nucleolus (type A nevus cells)	0	3	2
Small dense nuclei, resembling lymphocytes (type B cells)	0	2	2
Rounded, oval or elongated nucleus	5	3	0
Pseudo-inclusion	0	3	0
**Connective tissue/lamina propria**			
Maturation	0	2	2
Fibrosis	5	2	1
Neurotization (type C cells)	0	1	1
Mucinous degeneration	0	1	0
Giant cell	0	3	1
Fat accumulation	0	0	0
Angiomatous appearance	0	1	1
Herniation	0	1	0
Meissner’s corpuscle-like structure	0	0	0
Melanophages (pigment incontinence)	4	2	2
Ballooning cell	0	0	0
Dendritic and spindle-shaped cells parallel to the surface	7	0	0
Dendritic and spindle-shaped cells parallel to the surface, around nerves and blood vessels	1	0	0
Nevus cells around nerves and blood vessels	0	2	0
**Symmetry**	5	3	1

**Figure 1:**
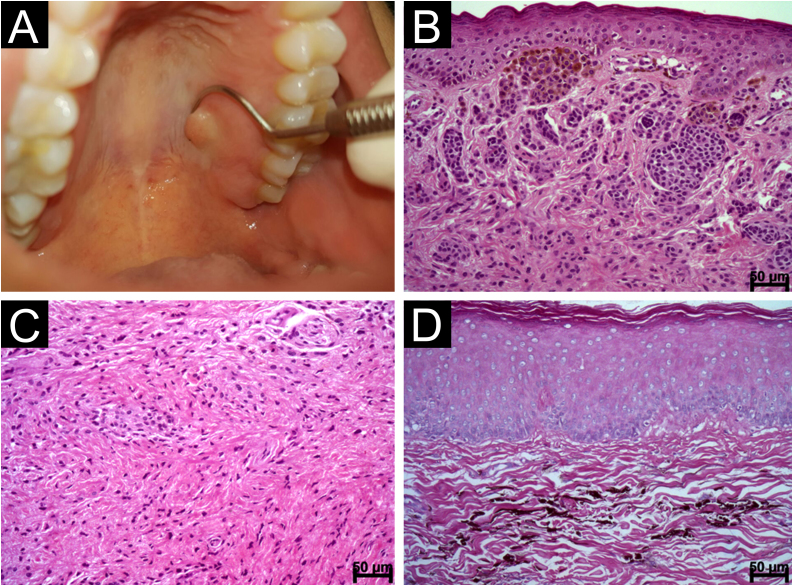
Intramucosal and blue nevi. (A) Nodular swelling covered with intact mucosa in the left region of the palate corresponding to an intramucosal nevus (B) Nests of pigmented nevus cells separated from the epithelial lining by an evident basement membrane. Note polygonal and epithelioid cells in the subepithelial region and the depth cells similar to lymphocytes (Hematoxylin & eosin, scale bar: 50 μ) (C) Intramucosal nevus showing deep-seated angled, spindle-shaped cells with neurotization; Type C (Hematoxylin & eosin, scale bar: 50 μ) (D) Blue nevus composed of spindle-shaped cells with long cytoplasm and dendritic cells containing brownish melanic pigment (Hematoxylin & eosin, scale bar: 50 μ).

**Figure 2:**
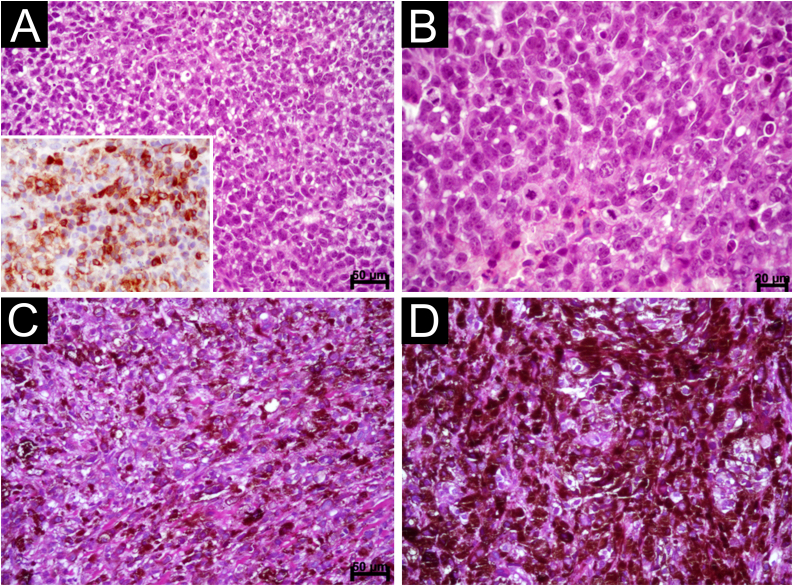
Oral Melanoma. (A) Diffuse proliferation of plasmacytoid and epithelioid cells containing hyperchromatic nuclei and absence of melanin pigment (Scale bar: 50 μ). Observe the immunostaining for melan TA (inset). (B) Detail of the previous image showing evident but not prominent nucleoli and mitotic figures (Scale bar: 20 μ) (C) Diffuse proliferation of epithelioid and spindle-shaped cells permeated by dark brown melanin pigment (Scale bar: 50 μ) (D) Area showing clotted pigment assuming the shape of epithelioid and spindle-shaped cells (Scale bar: 20 μ).

## Financial support

This study was supported by “Conselho Nacional de Desenvolvimento Científico e Tecnológico” (CNPq) and “Fundação de Amparo à Pesquisa no Estado da Bahia(FAPESB).

## Authors’ contributions

Izadora Fernanda Veiga De Jesus Costa: Study concept and design; Data collection, or analysis and interpretation of data; Writing of the manuscript or critical review of important intellectual content; Effective participation in the research guidance; Critical review of the literature; Final approval of the final version of the manuscript.

Jean Nunes dos Santos: Study concept and design; Data collection, or analysis and interpretation of data; Writing of the manuscript or critical review of important intellectual content; Effective participation in the research guidance; Critical review of the literature; Final approval of the final version of the manuscript.

Deyla Duarte Carneiro Vilela: Writing of the manuscript or critical review of important intellectual content; Critical review of the literature; Final approval of the final version of the manuscript.

Bruno Cunha Pires: Writing of the manuscript or critical review of important intellectual content; Critical review of the literature; Final approval of the final version of the manuscript.

Jener Gonçalves de Farias: Writing of the manuscript or critical review of important intellectual content; Critical review of the literature; Final approval of the final version of the manuscript.

Valéria Souza Freitas: Writing of the manuscript or critical review of important intellectual content; Critical review of the literature; Final approval of the final version of the manuscript.

## Conflicts of interest

None declared.
